# Image Representation Method Based on Relative Layer Entropy for Insulator Recognition

**DOI:** 10.3390/e22040419

**Published:** 2020-04-08

**Authors:** Zhenbing Zhao, Hongyu Qi, Xiaoqing Fan, Guozhi Xu, Yincheng Qi, Yongjie Zhai, Ke Zhang

**Affiliations:** 1 School of Electrical and Electronic Engineering, North China Electric Power University, Baoding 071003, China; nansbas@163.com (H.Q.); 18331129289@163.com (X.F.); qiych@126.com (Y.Q.); zhaiyongjie@ncepu.edu.cn (Y.Z.); zhangkeit@ncepu.edu.cn (K.Z.); 2 Hangzhou Institute, NetEase, Hangzhou 310052, China; hzxuguozhi@corp.netease.com

**Keywords:** image representation, insulator recognition, deep convolutional neural networks, relative layer entropy, vector of locally aggregated descriptors

## Abstract

Deep convolutional neural networks (DCNNs) with alternating convolutional, pooling and decimation layers are widely used in computer vision, yet current works tend to focus on deeper networks with many layers and neurons, resulting in a high computational complexity. However, the recognition task is still challenging for insufficient and uncomprehensive object appearance and training sample types such as infrared insulators. In view of this, more attention is focused on the application of a pretrained network for image feature representation, but the rules on how to select the feature representation layer are scarce. In this paper, we proposed a new concept, the layer entropy and relative layer entropy, which can be referred to as an image representation method based on relative layer entropy (IRM_RLE). It was designed to excavate the most suitable convolution layer for image recognition. First, the image was fed into an ImageNet pretrained DCNN model, and deep convolutional activations were extracted. Then, the appropriate feature layer was selected by calculating the layer entropy and relative layer entropy of each convolution layer. Finally, the number of the feature map was selected according to the importance degree and the feature maps of the convolution layer, which were vectorized and pooled by VLAD (vector of locally aggregated descriptors) coding and quantifying for final image representation. The experimental results show that the proposed approach performs competitively against previous methods across all datasets. Furthermore, for the indoor scenes and actions datasets, the proposed approach outperforms the state-of-the-art methods.

## 1. Introduction

**Figure 1:**
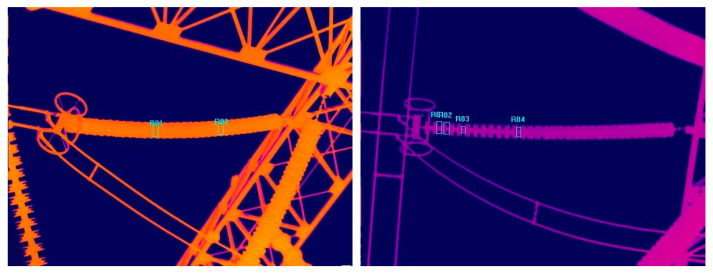
Infrared insulator images taken from a thermal image.

An insulator is an important part of the transmission line and power substation. Aside from its key role of providing electrical insulation and support lines [1], its running conditions directly affect the normal operation of the whole power grid. Ensuring the reliability and stability of transmission lines and power substations is an important part of smart grid inspection [2]. Temperature is an important indicator of insulator conditions [3]. The infrared imaging technology can detect problems with the insulation equipment under high voltage, high current and high temperature conditions. It is not subject to electromagnetic interference, which makes it a safe and reliable way to inspect electrical equipment. Its ability to assess the deterioration of insulators has been widely used [4,5]. However, the fault detection under extreme conditions has shown the need for improvement in the accuracy and efficiency for real-time intelligent insulator recognition methods [6,7]. Figure 1 shows insulators on a transmission line.

In the past few years, some progress has been made in insulator recognition based on machine learning. Wang et al. [8] proposed a novel insulator recognition method for images taken by unmanned aerial vehicles (UAVs). Because the UAV cameras provided highly cluttered backgrounds, a machine learning algorithm, support vector machine (SVM), was used as a classifier to distinguish the insulator from the cluttered background based on Gabor features. Wang et al. then expanded their research to develop an innovative background suppression method to remove the redundant background information as much as possible. Liu et al [9] proposed a method that initialized a six-level convolution neural network (CNN) and adjusted the training parameters to train the model. The obtained model was then applied to predict the candidate insulator position for insulator recognition. With the help of a non-maximum suppression algorithm and a linear fitting method, Liu et al. were able to pinpoint the exact location of an insulator. An insulator recognition method based on target recommendation and AdaBoost algorithm was proposed in [10], which can quickly locate insulators and improve the processing speed by changing the search window mechanism. In [11], a novel approach was proposed to inspect insulators with CNN. A CNN model with a multi-patch feature extraction method was applied to represent the status of insulators, and an SVM was trained based on these features. A thorough evaluation was given in [11] on this insulator status dataset of six classes by using on-site inspection videos.

Insulator feature representation based on deep learning is a novel recognition method. However, in practical applications, the labeled image data are scarce and expensive. Many researchers cannot obtain the required amount of labeled image data for CNN training and hence turn to the insulator feature representation method based on pretraining models instead.

## 2. Related Work

Deep convolutional neural networks (DCNNs) are state-of-the-art models for many computer vision tasks, such as image recognition, object detection, semantic segmentation and natural language processing [12,13]. Recent progress in computer vision has been driven by the use of large convolutional neural networks. Such networks benefit from alternating convolutional and pooling layers; the pooling layers serve to summarize small regions of the lower layer.

Since the remarkable progress made by AlexNet on ILSVRC 2012 [13], great efforts have been devoted to image recognition tasks with various machine learning skills. Most of these works focus on designing deeper network architectures, such as VGGNet [14], GoogleNet [15], Inception Network [16] and ResNet [17], which may contain hundreds of layers in their final forms. Nevertheless, they tend to have many layers and many neurons, resulting in high computational complexity. Several regularization techniques and data transformations have been designed to reduce the over-fitting effect of the deep network, such as multiscale cropping, dropout and a smaller convolutional kernel size. In addition, several optimization techniques have been proposed to reduce the computation amount of training networks to improve recognition performance, such as batch normalization (BN) and feature selection. Training a new deep model requires tuning millions of parameters, involving enormous training datasets and expensive computing hardware (e.g., the GPU). The whole optimization process usually takes a few days or even weeks, and the training process requires a lot of skills. In consequence, the need for a more efficient method was evident from the beginning.

Recently, a new method has been proposed that utilizes the deep feature activations extracted from a pretrained CNN model as a general feature extractor for images. DCNN activations are extracted for classifier training and have been successfully applied in various image recognition tasks. To get a generic representation, after a series of convolutional filtering and pooling, the neural activations from the first or second fully connected layers are usually extracted from a pretrained CNN model [18]. The research of [19] shows that the convolutional features can capture the local features and adjust the image structures, which can yield important cues for discriminating image recognition, whereas these features are mostly eliminated in the highly-compressed, fully-connected layers.

As noted in [20,21], the ensemble of features from different layers could boost the performance. A DCNN network contains multiple levels of image abstraction, which can be seen as rich semantic feature hierarchies. In [22], the features in the fourth or fifth convolutional layer were more robust due to their greater global scope, but the spatial locations of the features of the higher-level pattern were inaccurate (e.g., text or human faces). Confirming intuition, color and texture concepts dominated at lower layers, such as conv1 and conv2, while more object and part detectors emerged in conv5. Zeiler et al. [23] pointed out that the fifth layer activations reconstructed the visualization to make it look more like an input image. Later, in [24] it was pointed out that using sum-pooling to aggregate deep features on the last convolutional layer leads to better performance. The authors of [25] investigated several effective usages of CNN activations on both image retrieval and classification. In particular, they aggregated activations of each layer and concatenated them into the final representation, which achieved satisfactory results. The research of [26] and [27] also showed that visual recognition tasks make a considerable difference, which needs to be considered in the process of constructing a depth model. For example, during the construction of the action recognition model of [28], the adaptability of the model to a weak supervised dataset was taken into account.

**Figure 2:**
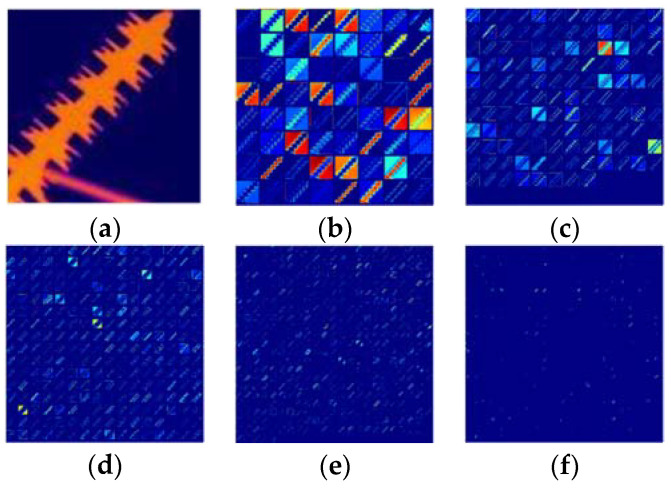
Infrared insulator images and their corresponding feature maps. The deep neural activations are highly related to the deformations of the input images. (**a**) Input image, (**b**) conv1 224 × 224 × 64, (**c**) conv2 112 × 112 × 128, (**d**) conv3 56 × 56 × 256, (**e**) conv4 28 × 28 × 512 and (**f**) conv5 14 × 14 × 512.

To generate deep feature descriptors, we looked to the vector of the locally aggregated descriptors (VLAD) aggregator [29,30], which built an image representation by aggregating residual errors for the grouped descriptors based on a locality criterion in the feature space. We visualize the feature maps of two insulators in Figure 2. The corresponding activations in the intermediate layers are shown in diverse patterns, which means the deep features are sensitive to rotation changes.

From the above research, we concluded that these approaches directly used the DCNN activations/descriptors and encoded them into a single representation without evaluating their suitability for different computer vision tasks. In light of this, we proposed a new concept of layer entropy and relative layer entropy. Then, an image representation method based on relative layer entropy (IRM_RLE) designed to excavate the most suitable convolutional layer for our image recognition was put forward. First, the infrared image of an insulator is fed into an ImageNet pretrained DCNN model, and deep convolutional activations are extracted. Then, the appropriate feature layer is selected by calculating the layer entropy and relative layer entropy of each convolutional layer. Finally, the number feature map according to the importance degree and the feature maps of the convolutional layer are vectorized and pooled by VLAD coding and then quantified for the final image representation. In IRM_RLE, a pretrained CNN model (which was not fine-tuned) was used for absolute supervision. We conducted extensive experiments on the infrared image dataset, which contained 4780 insulator images and 13,012 background images. We also accessed visible image datasets such as MIT-67 and Stanford 40 Actions. The experimental results not only verified the accuracy of our method, but also proved that our method can be applied to multi-modal images.

The rest of the paper is organized as follows: Section 3 presents the proposed method. Section 4 presents experimental results and a discussion. Conclusions are given in Section 5.

## 3. The Proposed Method

**Figure 3:**
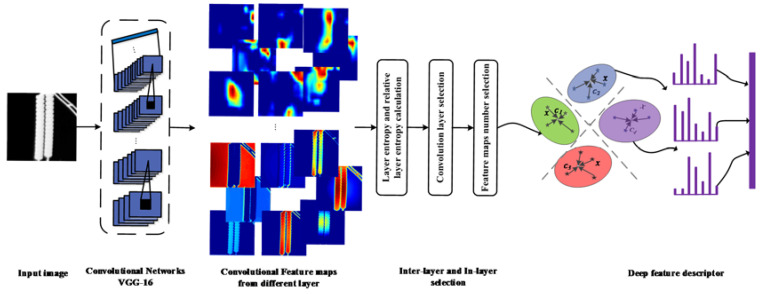
The global image descriptor generating framework. An infrared image of the insulator is fed into an ImageNet pretrained DCNN (deep convolutional neural networks.) model; then, deep convolutional activations are extracted. The appropriate feature layer is selected by calculating the layer entropy and relative layer entropy of each convolutional layer. The feature maps of the convolutional layer are vectorized and pooled by VLAD coding and quantifying. Finally, the final image representation is generated.

We propose the image representation method based on relative layer entropy (IRM_RLE). Our primary objective was to obtain compact and spatial invariant image representations. Instead of extracting features from the fully connected layers, we focused on the intermediate convolutional layers. Compared with activations from fully connected layers, the convolutional features are embedded with more spatial information. In this section, we introduce the DCNN model applied in our work and the convolutional feature maps in each layer. We then describe the deep convolutional layer and the in-layer feature map selection method. To encode the extracted DCNN features for classification, we adopt VLAD to aggregate the DCNN descriptors into a compact representation. The global image descriptor generated the framework is shown in Figure 3.

The following definitions are used herein: the “feature map” indicates the convolutional results of one channel; ”activations“ indicates feature maps of all channels in a convolutional layer; “descriptor” indicates the d-dimensional component vector of activations; and “conv5_3” refers to the last convolutional layer.

### 3.1. Deep Convolutional Neural Network Activations

We employed the VGG-16, also known as OxfordNet, which is a convolutional neural network structure developed by the Visual Geometry Group. The network consists of 13 convolutional layers and three fully connected layers. The convolutional layer consists of 3 × 3 small convolutional filters and five max-pooling layers. This network was the winner of the 2014 ImageNet Large Scale Visual Recognition Challenge (ILSVRC2014). Today, VGG is still considered an outstanding visual model, although its performance has actually been behind Inception and ResNet. The detailed parameters of the network architecture are listed in Table 1.

On each convolutional layer *l*, a convolutional operation is conducted on its *M_l−_*_1_ input maps from previous layer *l*−1 with a filter of size *k_l_* × *k_l_*. The resulting output is the summations of the responses with a non-linear function:

(1) $$F_{j}^{l}=f\left(\sum_{i\in M_{j}}F_{i}^{l-1}*W_{ij}^{l}+b_{j}^{l}\right)$$

where *l* indicates the layer; *F_j_*^l^ and *F_j_*^l−1^ are the activations from layer *l* and layer *l*−1 with filter size *k_l_* × *k_l_*, respectively. The term *b* indicates the bias, and *f*(·) is the ReLU (rectified linear unit) function:

(2) $$f\left(x_{i}\right)=\{\begin{array}{c} x_{i},\begin{array}{c} x_{i}>0 \end{array}\\ 0,\begin{array}{c} x_{i}\leq 0 \end{array} \end{array}$$

A feature map reveals the distribution of the neural activity [31]. Given an input image *I* with size *H* × *W*, the activations from a convolutional layer are formulated as a third-order tensor *T* with size *h* × *w* × *d*, which includes a set of 2D feature maps *S* = (*S*_1_, *S*_2_, …, *S_n_*)(*n* = 1, …, *d*). S*_n_* (size *h* × *w*) is the *n*-th feature map of the corresponding channel, as illustrated in Table 1. By applying the pretrained VGG-16 model, we extract the feature maps from a low-level convolutional layer to a high-level convolutional layer.

**Figure 4:**
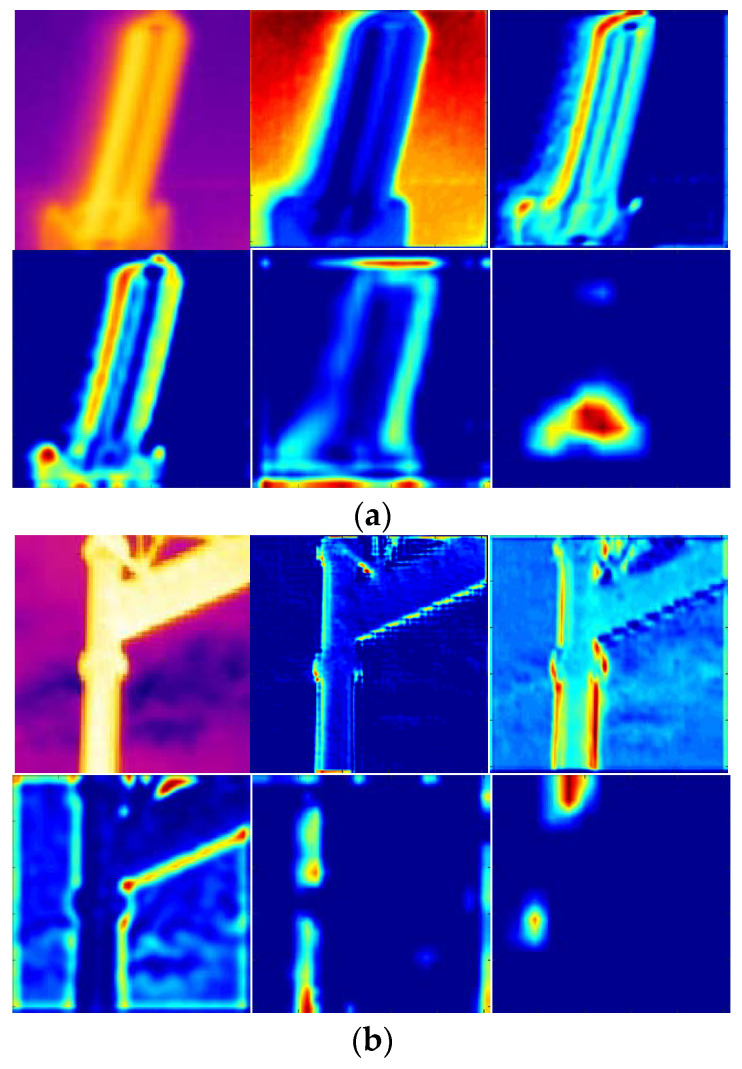
Visualizations of randomly sampled feature maps from intermediate convolutional layers of conv1_2, conv2_2, conv3_3, conv4_3 and conv5_3 images from an infrared insulator dataset. (**a**) Insulator and (**b**) background.

In Figure 4, we randomly selected an infrared insulator image and its background image from our infrared insulator dataset. Then we visualized the feature maps from different convolutional layers. From the visualization of these feature maps, we can see that the rich semantic feature hierarchies with lower convolutional layers captured local features with detailed spatial information, and the features in the higher layers were more abstract with rich semantic information, which is very powerful at distinguishing different classes.

### 3.2. Deep Convolutional Layer Selection

The existing selection approach uses DCNN activations directly and encodes them into a representation without evaluating whether it is the most suitable feature representation method for different classification and recognition tasks. In view of this, we proposed a method to correct this approach. We employed the image entropy; i.e., the image representation method based on relative layer entropy, which is designed to determine which convolutional layer is the most suitable one for different image recognition tasks. The image entropy was expressed as the average number of bits in the image gray level set, and it can describe the average amount of information for the image source. First, we proposed a new concept of convolutional layer entropy. We defined the layer entropy as the sum of the image entropy of all the feature maps from the convolutional layer. To mine the neuron’s response pattern, the feature map elements were normalized to a range from 0 to 255, and the image entropy of the feature map can be calculated by Equation (3). For the convolutional layer l, the number of output feature maps is M, and the layer entropy of each convolutional layer is calculated by Equation (4).

(3) $$H\left(S\right)=-\sum_{n=0}^{255}p_{n}\log_{2}\left(p_{n}\right)$$

where, *p_n_* represents the probability of the gray-scale value *n* emerging within the image, and

(4) $$H_{l}=\sum_{i=1}^{M}H(S)$$

We found that layer entropy is different due to the image entropy of the input image’s divergence. To balance this difference, we raised the concept of relative layer entropy, which is the ratio of the entropy of a convolutional layer to the image entropy of the input image and the product of the number of feature maps. First, the image entropy *H*(*S_I_*) of the input image is calculated by Equation (3), and the relative layer entropy of each convolutional layer is calculated using Equation (5).

(5) $$H=H_{l}/\left[H(S_{I})*M\right]$$

The standard deviation of a layer entropy can be acquired as:

(6) $$\sigma \left(H\right)=\sqrt{\frac{1}{G}\sum_{i=1}^{G}{\left(H_{l}-\mu \right)}^{2}}$$

where *G* is the total number of convolutional layers of the deep model and *µ* is the mean value. By combining the two quantification methods, the deep convolutional layer selection can be described as:

(7) $$L=H-\sigma (H)/\left[H(S_{I})*M\right]*l$$

For all layers of the deep neural network, the layers with the smallest *L* value were selected to make up the final feature representative layer.

### 3.3. In-Layer Feature Map Selection

From AlexNet to ResNet, the DCNNs for visual recognition have grown deeper in the quest for higher classification accuracy. Depth has been shown to be important to high discrimination ability [32]. However, the width of layers (the number of units per layer) has been less explored. One reason is that increasing the number of convolutional units in a layer significantly increases computational cost while yielding only tiny improvements in classification accuracy. Nevertheless, some recent work [33] shows that a carefully designed network can achieve classification accuracy, superior to the commonly used thin and deep counterparts.

To explore how the width of layers affects interpretability of CNNs, we did a preliminary experiment to test the influence of the width on the emergence of interpretable classification. According to the visualization result of the convolutional layer feature map, we found that some feature graphs contain redundant information and have great influence on the classification result. The research shows that a feature map is usually sparse and some semantic regions are indicated. To remove the redundant information, we selected the number of convolutional layer feature maps.

Suppose *x_i_^l^* is the information contained in the *l*-th feature map of the *i*-th layer; *x_i_^max^* is the maximum information in the *i*-th layer effective information; *x* is the ratio between *x_i_^l^* and *x_i_^max^*; and *y_i_^l^* is the useful information contained in the *l*-th feature map of the *i*-th layer.

$$\underset{x\to 1}{\lim}p(y_{i}^{l}|x)=\underset{x\to 1}{\lim}\frac{p(x,y_{i}^{l})}{p(x)}=\underset{x\to 1}{\lim}\frac{p(x)\cdot p(y_{i}^{l})}{p(x)}=p(y_{i}^{l})$$

$$x_{2}<x_{1}\to 1\Rightarrow p(y_{2})<p(y_{1})$$

The more information contained in a feature map, the more useful information it contains. Therefore, we employed the feature map ranking strategy based on the activation patterns of neurons and adopted it for feature map selection. The first step was to quantify the importance of feature maps. We used the classic image entropy as the quantification method. Thus, the entropy of a feature map *S_j_* can be computed as:

(8) $$H\left(S_{j}\right)=-\sum_{n=0}^{255}p_{n}\log_{2}\left(p_{n}\right)$$

**Figure 5:**
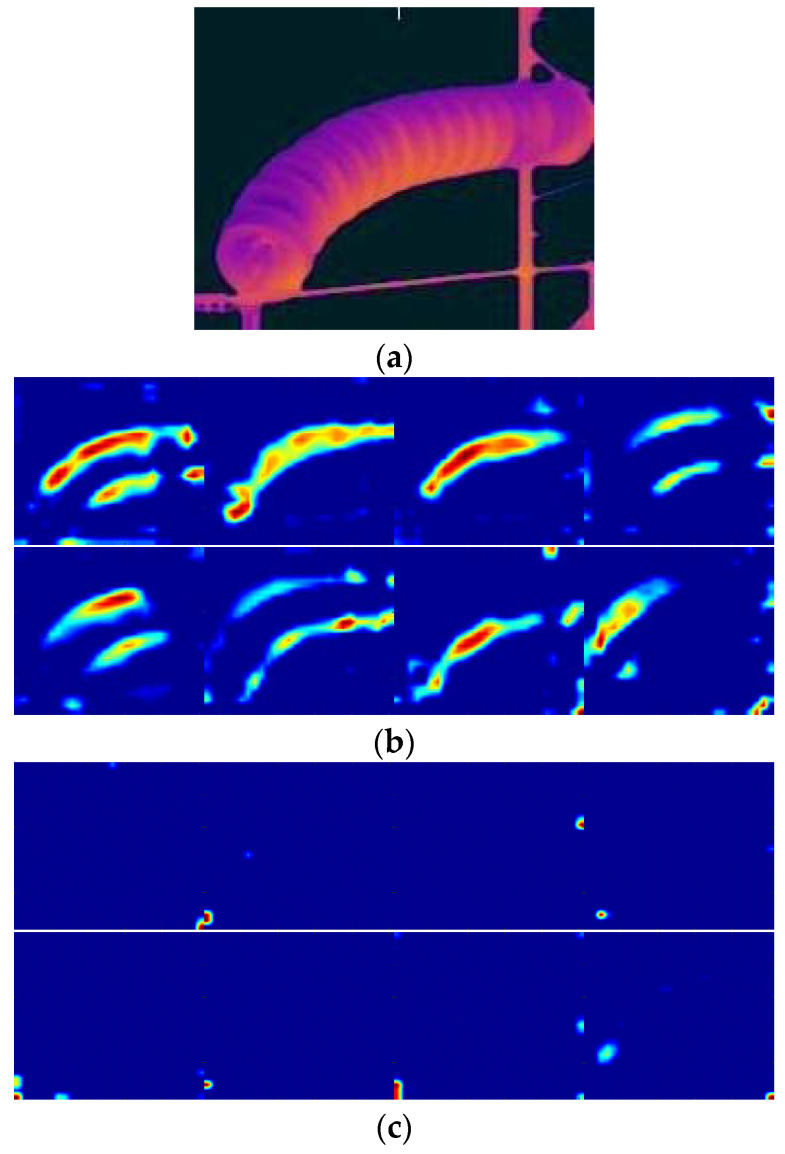
Ranking feature maps with high importance degree scores from conv4_3 layer of the insulator image. (**a**) Input image, (**b**) top-ranking images and (**c**) the lower ranking images.

**Figure 6:**
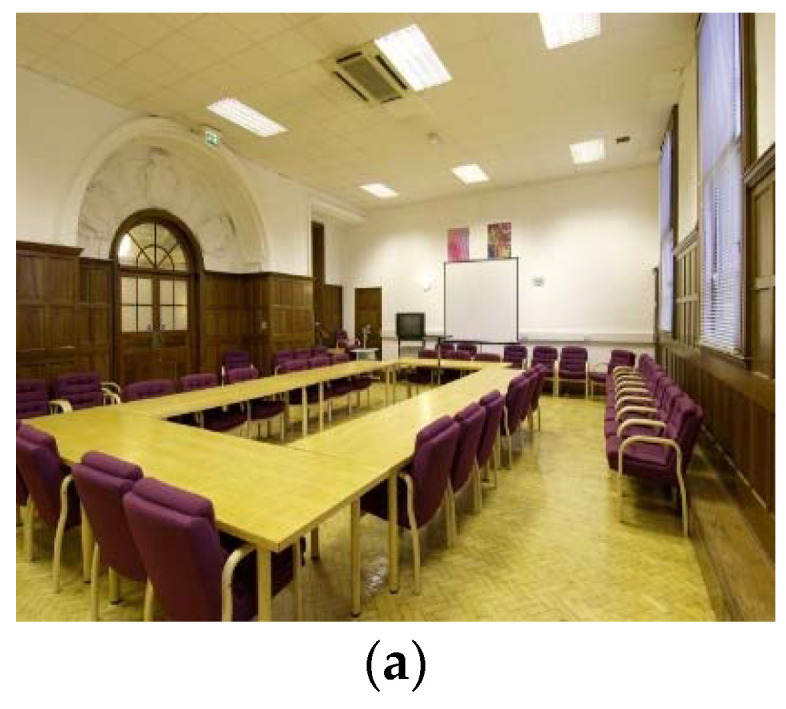

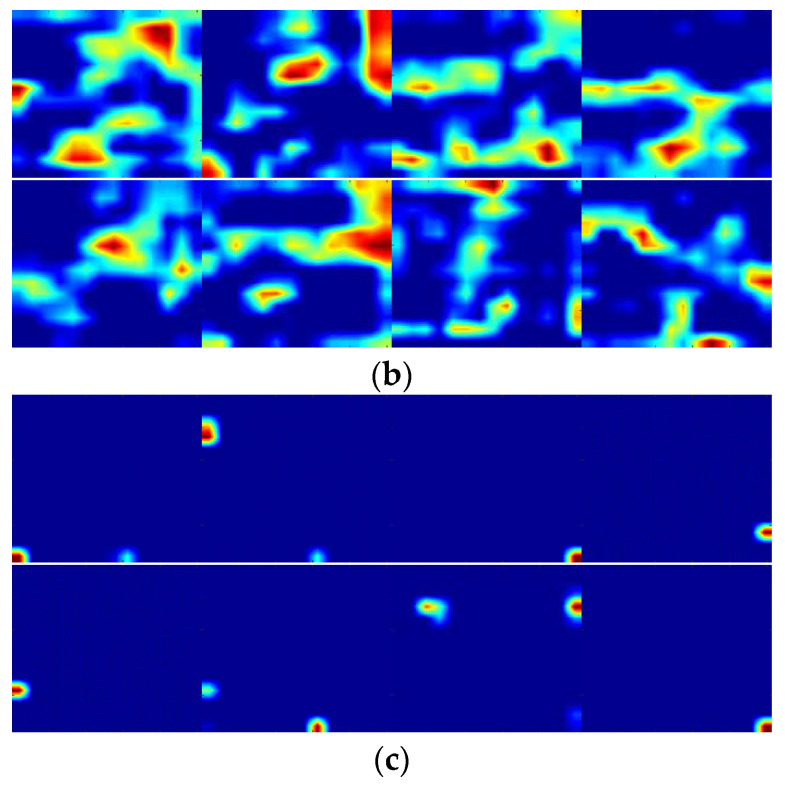
Ranking feature maps with high importance degree scores from conv5_2 layer of the scene image. (**a**) Input image, (**b**) the top-ranking images and (**c**) the lower ranking images.

The standard deviation of a feature map *S_j_* can be acquired as:

(9) $$\sigma \left(S_{j}\right)=\sqrt{\frac{1}{N}\sum_{j=1}^{N}{\left(x_{j}-\mu \right)}^{2}}$$

where *x_j_* means the value of the *j*-th element and *μ* is the mean value. By combining the two quantification methods, the importance degree of convolutional feature map *S_j_* can be presented as:

(10) $$K\left(S_{j}\right)=H\left(S_{j}\right)+\lambda \sqrt{\sigma \left(S_{j}\right)}$$

where *λ* is empirically set at 0.01. We then computed the importance degree score of the feature map extracted. Based on the computed importance degree score, we sorted all the feature maps from the same layers. Part of the sorting results of insulators with conv4_3 layer and the scene images with conv5_2 layer are demonstrated in Figure 5 and Figure 6, respectively.

We selected the top-*Q* feature maps from *M* convolutional feature maps. The selected feature maps contain most of the useful information, while the depth is half that of the unselected feature tensor. We then stacked all the selected feature maps as the newly generated tensor, and applied the new tensor for the following feature pooling.

### 3.4. Deep Convolutional Descriptor Aggregation

Intuitively, we applied VLAD for the IRM_RLE feature generation. We first performed the deep convolutional layer and in-layer feature map selection, and the codebook *C* = {*c*_1_, *c*_2_, …, *c_k_*} was generated by *k*-means clustering on the selected deep feature maps from the deep convolutional layer as described in Section 3.2. When the clustering is finished, the centers are assigned as *k* visual words. The codebook is a *k* × *D* matrix, composed of *k* visual words with dimension *D*.

Given an input image *I*, first, the selected feature map can be seen as a set of deep descriptors *X* = (*x*_1_, *x*_2_, …, *x_n_*). Then, each descriptor *x_n_* is associated with its nearest visual word *c_n_* = *NN*(*x_n_*), and *NN* indicates the nearest neighbor search. The nearest *c*(*x_n_*) can be indexed by Equation (11), where *d*|•| denotes the distance between two features.

(11) $$c\left(x_{n}\right)=\arg\underset{c_{i}}{\min}d\left|c_{i},x_{n}\right|$$

VLAD encodes feature *x_n_* by considering the residuals:

(12) $$v_{i}=\sum_{x_{n}:c(x)=c_{i}}x_{n}-c_{i}$$

Then the residuals are stacked together to obtain the final vector:

(13) $$v(I)=\left[v_{1},\ldots ,v_{i},\ldots ,v_{k}\right]$$

The ensemble of features from different layers can boost the performance. Thus, we applied a set of convolutional layers for compact image representation generation, and we considered all layers as contributing equally to the final representation. We distributed a same weight to each layer employed. The method is summarized in Algorithm 1.
**Algorithm 1:** Deep convolutional feature representation generation.Input: Pretrained model, image *I* Output: IRM_RLE feature vector *V*(*I*) Procedure: 1. Extract deep feature maps from layer *l*, *S* = [*S*_1_,…,*S_i_*,…,*S_n_*] 2. Layer entropy and relative layer entropy calculation 3. Deep convolutional layer selection 4. Compute importance degree of each feature maps $K(S_{i})=H(S_{i})+\lambda \sqrt{\sigma (S_{i})}$ 5. Select top-ranked *Q* feature maps 6. Extract deep descriptors from the feature map tensor *X* = (*x*_1_, *x*_2_, …, *x_n_*) 7. *k*-means clustering for codebook *C* = {*c*_1_, *c*_2_, …, *c_k_*} 8. Aggregating deep descriptors for *i* = 1 to *n* do  *t* = index argmin *d*|*c_j_*, *x_i_*|, *j* ∈ {1, 2, …, *k*}  v’_t_ = v’_t_ + (x_i_ − c_t_)  end for $v(I)=\left[v_{1},\ldots ,v_{i},\ldots ,v_{k}\right]$ 9. $V(I)=\left[v_{i}^{1}*W_{i}^{1},\ldots ,v_{i}^{l}*W_{i}^{l}\right]$ Return: *V*(*I*)

## 4. Experiments

We evaluated our proposed method based on our current infrared insulator image dataset and two publicly available visible image datasets called MIT Indoor67 and Stanford 40 Actions.

### 4.1. Dataset and Experiment Setup

**Figure 7:**
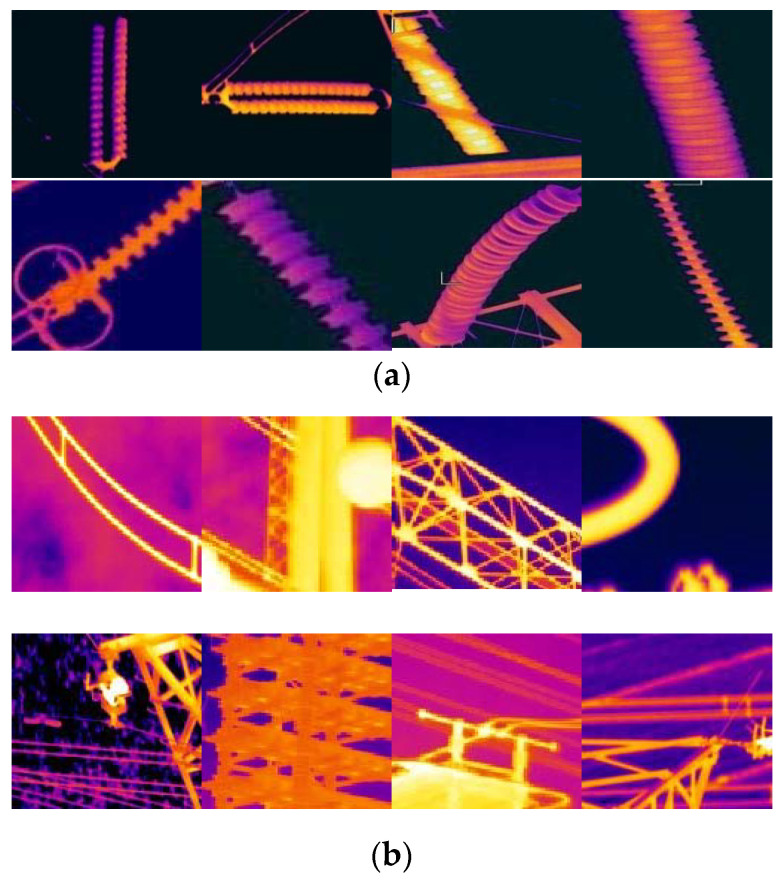
Sample images of infrared insulator dataset. (**a**) Insulators and (**b**) background.

Due to the difficulty of obtaining insulator infrared images and the absence of public infrared image datasets, we used lots of infrared images collected from our insulator inspection system to build the insulator infrared image datasets. The infrared insulator image dataset consists of 4780 insulator images and 13,012 background images. The insulator images were manually cropped from the original images taken in the power substations and transmission lines, which varied from 110 to 500 kV in levels. We divided the dataset into two parts: 30% for training and the remaining 70% for testing. All the training samples were labeled as “insulator" and “background." Examples of the images in our infrared insulator dataset are shown in Figure 7.

The DCNN model we employed is VGG-16 with 13 convolutional layers pretrained on ImageNet. We did not fine-tune the arbitrary dataset. SVM was chosen for classification of insulators which is widely used because of its ability to avoid over-fitting and its excellent performance on small datasets.

### 4.2. Results on the Infrared Insulator Dataset

In this experiment, we simply extracted features from the conv4_3 according to the selected results based on IRM_RLE and selected the feature map number for descriptor aggregation. In VLAD encoding, the number of centers *k* determines the dimension of the final feature. To save time and reduce the quantity of calculations, we fixed the number of VLAD centers to 100 in our infrared insulator experiments to obtain good performances. The performance parameter of the classifier is calculated as:

(14) $$Accuracy=\frac{TP+TN}{TP+TN+FP+FN}$$

where *TP* is the true positive, *TN* is the true negative, *FP* is the false positive and *FN* is the false negative. The proposed deep feature vector generation was conducted for each image in the training set, and an SVM classifier was trained for classification. To evaluate the performances, we extracted the feature maps from all convolutional layers and applied VLAD encoding to aggregate these feature maps. Different features were compared, and the classification accuracies of all the convolutional layers are presented in Table 2.

**Figure 8:**
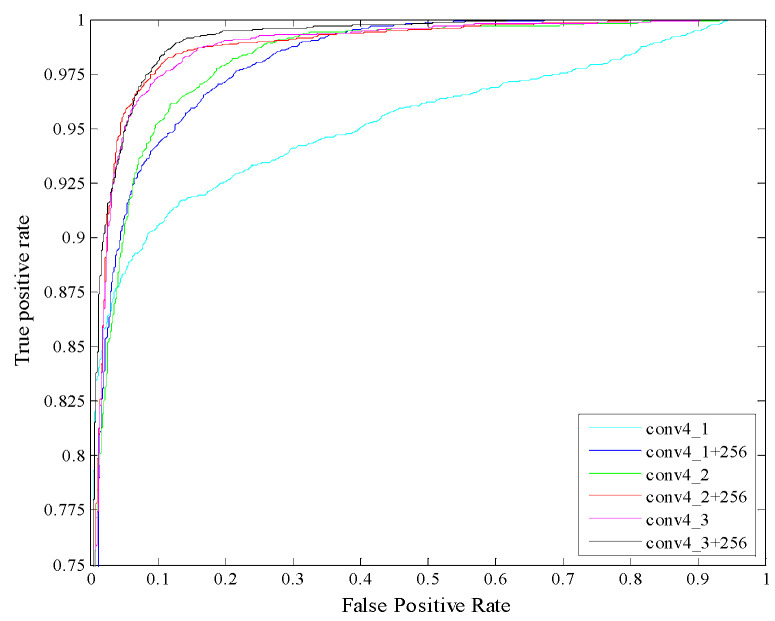
ROC (receiver operating characteristic) of different layers and different numbers of feature maps. Conv4_1 represents extraction of all the feature maps in conv4_1 for descriptor aggregation. Conv4_1+256 represents extraction of half of the feature maps in conv4_1 for descriptor aggregation.

When the distribution of positive and negative samples in the test set changes, the ROC (receiver operating characteristic) curve remains unchanged. To show the necessity of selecting the deep convolutional layers and the number of intra-layer feature maps, we used the ROC curve to illustrate the experimental results in Figure 8.

As can be seen from Table 2, the performance of conv4_3 is the best, corresponding to our IRM_RLE method selection results. The experiment results show that the deep convolutional layer with the richest semantic information is not the most suitable layer for classification and recognition. For the single-objective classification problem of insulators, the relatively low-level convolutional layer can achieve a better effect and take less time.

In ROC space, the more directed the ROC curve is to the upper left, the better effect is. As can be seen from Figure 8, the cyan curves represent experimental results of conv4_1 while the blue curve represents the experimental result of extracting half of the feature maps in conv4_1 for descriptor aggregation. The experimental results of the blue curve are obviously better than the cyan curve. The blue, red and black curves represent the experimental results that extract half of the feature maps in conv4_1, conv4_2, and conv4_3 for descriptor aggregation, respectively. The black curve works best, corresponding to our classification results shown in Table 2.

### 4.3. Evaluation Experiment on Public Datasets

**Figure 9:**
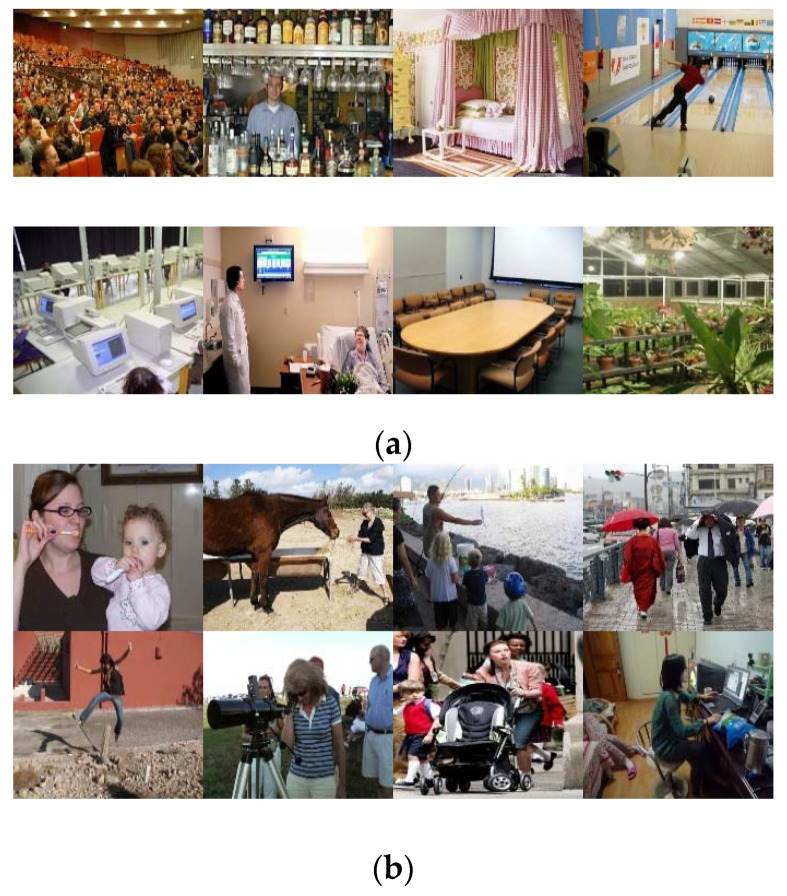
Sample images of MIT Indoor67 and Stanford 40 Actions. (**a**) MIT Indoor67 and (**b**) Stanford 40 Actions.

In our evaluation experiments, we evaluated the proposed approach on two public visible image datasets, the MIT Indoor67 [34] and the Stanford 40 Actions (Stanford-40) [35]. The MIT Indoor67 is a unique large and diverse database for indoor scene recognition. This database consists of 67 indoor categories covering a wide range of domains, and contains 15,620 images in total. The standard training/test split for the Indoor dataset has 80 training and 20 test images per class. Sample images are shown in Figure 9a.

Stanford 40 Actions contains images of humans performing 40 different classes of actions, including visually-challenging cases such as “fixing a bike” versus “riding a bike” and “phoning” versus “texting a message.” The number of samples per class varies from 180 to 300, for a total of 9532 images. A standard training/test split is made available by the authors on their website, selecting 100 images from each class for training and leaving the remainder for testing. Sample images are shown in Figure 9b.

In the VLAD encoding, we fixed the number of VLAD centers to 256 in our experiments to obtain good performances. We first applied component-wise *l*_2_ normalization on each feature vector *v_k_* and then used global *l*_2_ normalization on the VLAD descriptor *V*(*I*). In image classification, the generated feature dimension was usually very high. Thus, we applied a one-versus-all multi-class linear SVM as the classifier. The LIBLINEAR [36] implementation was used in our experiments. We set parameter *C* to 0.01, and used open source libraries such as VLFeat, Caffe and LIBLINEAR.

In the MIT Indoor67 experiment, the performance of conv5_2 layer was the best, while conv5_1 layer came in second and conv5_3 layers was third according to our calculation results using the IRM_RLE method. In the Stanford 40 Actions experiment, the performance of conv5_1 layer was the best; conv5_2 layer came in second and conv5_3 layers came in third according to our calculation results using the IRM_RLE method. The classification results are shown in Table 3 and Table 4. To improve accuracy, we applied a set of convolutional layers (conv5_1, conv5_2 and conv5_3) for compact image representation generation. We considered all the layers as contributing equally to the final representation.

In Table 3, VLAD Multi-scale is the pooling baseline in [37], and VLAD level 2 was formed by extracting activations from 128 × 128 patches. We used VLAD to pool them with a codebook of 100 centers. The MOP-CNN (multi-scale orderless pooling for CNN) is the method proposed for combining several levels. CL+CNN-Jitter refers to the cross-convolutional-layer pooling proposed in [39]. Table 4 shows the results of the methods proposed in [40] after training on the various Places-CNNs; then, the final output layer of each network was used to classify the test set images.

From Table 3 and Table 4, we can see that our method achieved good performance with respect to accuracy and calculation quantity. The DCNN based methods outperformed the traditional methods, which are based on hand-crafted features and the end-to-end training method. Directly extracting the activations from the fully connected layer for SVM training is not the best method. From our point of view, the activations from the fully-connected layers are sensitive to spatial transformations, and the images in the MIT Indoor67 and Stanford 40 Actions share a large number of global transformations. Discovering the most important information from the convolutional layers can be a useful strategy for better feature extraction. The IRM_RLE method provided direction for the deep convolutional layer selection, saving both time and computation.

## 5. Conclusions

Deep convolutional neural networks are the state-of-the-art approaches in the computer vision and pattern recognition field, especially in image classification tasks. Inspired by the recent success of deep learning, we proposed the image representation method based on relative layer entropy for infrared insulator recognition. By calculating the relative layer entropy to select the most suitable convolutional layer and extracting the feature maps for aggregation to form feature representation, some good results were obtained. In this paper, we propose a new concept, the layer entropy and relative layer entropy, for infrared insulator recognition. DCNNs have a powerful ability to learn and represent features in a more distinctive way. Thus, time is saved by the absence of the need for fine-tuning in the infrared insulator recognition and scene classification task. However, the image representation method based on relative layer entropy has a higher recognition accuracy for infrared insulator images than for public visible light images, which means that the method has room for performance improvements on public, visible light image dataset recognition tasks. For example, in order to improve the recognition accuracy, the more optimized network structure design based on layer entropy can be studied.

In the future, the method presented in this paper can be applied to the recognition and detection of transmission line defects and provide excellent feature expression calculation.

## Figures and Tables

**Table 1:** Details of the feature maps from VGG-16.

Layer	Output Size		
	Width	Height	Depth
conv1_1	224	224	64
conv1_2	224	224	64
conv2_1	112	112	128
conv2_2	112	112	128
conv3_1	56	56	256
conv3_2	56	56	256
conv3_3	56	56	256
conv4_1	28	28	512
conv4_2	28	28	512
conv4_3	28	28	512
conv5_1	14	14	512
conv5_2	14	14	512
conv5_3	14	14	512

**Table 2:** Details of the classification results of different intermediate layers. (codebook size = 100).

Depth	Size of the Feature Maps	Descriptor Length	Accuracy (%)
conv2_1	112 × 112 × 128	1,254,400	0.9322
conv2_2	112 × 112 × 128	1,254,400	0.9839
conv3_1	56 × 56 × 256	313,600	0.9869
conv3_2	56 × 56 × 256	313,600	0.9921
conv3_3	56 × 56 × 256	313,600	0.9930
conv4_1	28 × 28 × 512	78,400	0.9869
conv4_2	28 × 28 × 512	78,400	0.9904
conv4_3	28 × 28 × 512	78,400	0.9942
conv5_1	14 × 14 × 512	19,600	0.9883
conv5_2	14 × 14 × 512	19,600	0.9897
conv5_3	14 × 14 × 512	19,600	0.9921

**Table 3:** Classification results on MIT Indoor67.

Method	Accuracy
SPM	34.40%
FV+Bag of parts	63.18%
DPM	37.60%
VLAD Multi-scale [37]	66.12%
VLAD level 2 [37]	65.52%
MOP-CNN [37]	68.88%
Fine-tuning [38]	66.00%
CNN-FC-SVM	58.40%
CL+CNN-Jitter [39]	71.50%
IRM_RLE	68.88%
IRM_RLE	70.52%
IRM_RLE	66.87%
IRM_RLE [5_1, 5_2, 5_3]	71.87%

**Table 4:** Classification results on Stanford 40 Actions.

Method	Accuracy
Sparse Bases	45.7%
Color Action Recognition	51.9%
Multiple Instance Learning	55.6%
Very Deep Network	71.7%
Action-Specific Detectors	75.4%
Places365-VGG [40]	49.20%
Places205-VGG [40]	53.33%
ImageNet-VGG [40]	66.63%
Hybrid1365-VGG [40]	68.11%
IRM_RLE 5-1	70.05%
IRM_RLE 5-2	69.50%
IRM_RLE 5-3	69.38%
IRM_RLE [5-1, 5-2, 5-3]	72.23%

## References

[1] Jiang H., Jin L., Yan S. (2015). Recognition and fault diagnosis of insulator string in aerial images. J. Mech. Electr. Eng..

[2] Zhao Z., Qi H., Qi Y., Zhang K., Zhai Y., Zhao W. (2020). Detection Method Based on Automatic Visual Shape Clustering for Pin-Missing Defect in Transmission Lines. IEEE Trans. Instrum. Meas..

[3] Nguyen V.N., Jenssen R., Roverso D. (2018). Automatic autonomous vision-based power line inspection: A review of current status and the potential role of deep learning. Int. J. Electr. Power Energy Syst..

[4] Zhao Z., Xu G., Qi Y. (2016). Representation of binary feature pooling for detection of insulator strings in infrared images. IEEE Trans. Dielectr. Electr. Insul..

[5] Zhaohui L., Weiping F., Zihui Y., Yunpeng L., Jiangwei W., Shaotong P. Insulator identification method based on infrared image. Proceedings of the 2017 IEEE International Conference on Smart Grid and Smart Cities (ICSGSC).

[6] Fang J., Wang J., Yang L., Wang G., Han J., Guo S. (2016). Detection method of porcelain insulator contamination grade based on infrared-thermal-image. Trans. Chin. Soc. Agric. Eng..

[7] Shen-Pei Z., Xi L., Bing-Chen Q., Hui H. (2017). Research on Insulator Fault Diagnosis and Remote Monitoring System Based on Infrared Images. Procedia Comput. Sci..

[8] Wang X., Zhang Y. Insulator identification from aerial images using Support Vector Machine with background suppression. Proceedings of the 2016 International Conference on Unmanned Aircraft Systems (ICUAS).

[9] Liu Y., Yong J., Liu L., Zhao J., Li Z. The method of insulator recognition based on deep learning. Proceedings of the 2016 4th International Conference on Applied Robotics for the Power Industry (CARPI).

[10] Wu Y. (2016). Research on Insulator Recognition Methods in Aerial Images Based on Machine Learning.

[11] Zhao Z., Xu G., Qi Y., Liu N., Zhang T. Multi-patch deep features for power line insulator status classification from aerial images. Proceedings of the 2016 International Joint Conference on Neural Networks (IJCNN).

[12] Girshick R., Donahue J., Darrell T., Malik J., Malik J. Rich Feature Hierarchies for Accurate Object Detection and Semantic Segmentation. Proceedings of the 2014 IEEE Conference on Computer Vision and Pattern Recognition.

[13] Krizhevsky A., Sutskever I., Hinton G.E. (2017). Pdf ImageNet classification with deep convolutional neural networks. Commun. ACM.

[14] Simonyan K., Zisserman A. Very deep convolutional networks for large-scale image recognition. Proceedings of the International Conference on Learning Representations (ICLR).

[15] Szegedy C., Liu W., Jia Y., Sermanet P., Reed S., Anguelov D., Erhan D., Vanhoucke V., Rabinovich A. Going deeper with convolutions. Proceedings of the 2015 IEEE Conference on Computer Vision and Pattern Recognition (CVPR).

[16] Szegedy C., Vanhoucke V., Ioffe S., Shlens J., Wojna Z. Rethinking the Inception Architecture for Computer Vision. Proceedings of the 2016 IEEE Conference on Computer Vision and Pattern Recognition (CVPR).

[17] He K., Zhang X., Ren S., Sun J. Deep Residual Learning for Image Recognition. Proceedings of the 2016 IEEE Conference on Computer Vision and Pattern Recognition (CVPR).

[18] Alrjebi M.M., Pathirage N., Liu W., Li L. (2017). Face recognition against occlusions via colour fusion using 2D-MCF model and SRC. Pattern Recognit. Lett..

[19] Guo S., Huang W., Wang L., Qiao Y. (2017). Locally Supervised Deep Hybrid Model for Scene Recognition. IEEE Trans. Image Process..

[20] Hariharan B., Arbelaez P., Girshick R., Malik J. Hypercolumns for object segmentation and fine-grained localization. Proceedings of the 2015 IEEE Conference on Computer Vision and Pattern Recognition (CVPR).

[21] Kulkarni P., Zepeda J., Jurie F., Pérez P., Chevallier L., Praveen K. Hybrid multi-layer deep CNN/aggregator feature for image classification. Proceedings of the 2015 IEEE International Conference on Acoustics, Speech and Signal Processing (ICASSP).

[22] Yosinski J., Clune J., Nguyen A., Fuchs T., Lipson H. Understanding Neural Networks through Deep Visualization. Proceedings of the International Conference on Machine Learning (ICML).

[23] Zeiler M.D., Fergus R. Visualizing and understanding computer vision. Proceedings of the European Conference on Computer Vision (ECCV).

[24] Babenko A., Lempitsky V. Aggregating Deep Convolutional Features for Image Retrieval. Proceedings of the IEEE International Conference on Computer Vision (ICCV).

[25] Zheng L., Zhao Y., Wang S., Wang J., Tian Q. (2016). Good Practice in CNN Feature Transfer. arXiv.

[26] Zhang X.-Y., Li C., Shi H., Zhu X., Li P., Dong J. (2020). AdapNet: Adaptability Decomposing Encoder-Decoder Network for Weakly Supervised Action Recognition and Localization. IEEE Trans. Neural Networks Learn. Syst..

[27] Zhang X.-Y., Wang S., Zhu X., Yun X., Wu G., Wang Y. (2015). Update vs. upgrade: Modeling with indeterminate multi-class active learning. Neurocomputing.

[28] Zhang X.-Y., Shi H., Li C., Zheng K., Zhu X., Duan L. Learning Transferable Self-Attentive Representations for Action Recognition in Untrimmed Videos with Weak Supervision. Proceedings of the AAAI Conference on Artificial Intelligence.

[29] Jégou H., Douze M., Schmid C., Perez P. Aggregating local descriptors into a compact image representation. Proceedings of the 2010 IEEE Computer Society Conference on Computer Vision and Pattern Recognition.

[30] Jégou H., Perronnin F., Douze M., Sanchez J., Perez P., Schmid C. (2011). Aggregating Local Image Descriptors into Compact Codes. IEEE Trans. Pattern Anal. Mach. Intell..

[31] Wei X.-S., Luo J.-H., Wu J., Zhou Z.-H. (2017). Selective Convolutional Descriptor Aggregation for Fine-Grained Image Retrieval. IEEE Trans. Image Process..

[32] Bau D., Zhou B., Khosla A., Oliva A., Torralba A. Network Dissection: Quantifying Interpretability of Deep Visual Representations. Proceedings of the 2017 IEEE Conference on Computer Vision and Pattern Recognition (CVPR).

[33] Zagoruyko S., Komodakis N., Wilson R.C., Hancock E.R., Smith W.A.P., Pears N.E., Bors A.G. Wide Residual Networks. Proceedings of the British Machine Vision Conference 2016.

[34] Quattoni A., Torralba A. Recognizing indoor scenes. Proceedings of the IEEE Conference on Computer Vision and Pattern Recognition (CVPR).

[35] Yao B., Jiang X., Khosla A., Lin A.L., Guibas L., Fei-Fei L. Human action recognition by learning bases of action attributes and parts. Proceedings of the 2011 International Conference on Computer Vision.

[36] Fan R.E., Chang K.W., Hsieh C.J., Wang X.R., Lin C.J. (2008). LIBLINEAR: A library for large linear classification. J. Mach. Learn. Res..

[37] Gong Y., Wang L., Guo R., Lazebnik S. (2014). Multi-scale Orderless Pooling of Deep Convolutional Activation Features. Proceedings of the Lecture Notes in Computer Science.

[38] Azizpour H., Razavian A.S., Sullivan J., Maki A., Carlsson S. From generic to specific deep representations for visual recognition. Proceedings of the 2015 IEEE Conference on Computer Vision and Pattern Recognition Workshops (CVPRW).

[39] Liu L., Shen C., Hengel A.V.D. The treasure beneath convolutional layers: Cross-convolutional-layer pooling for image classification. Proceedings of the 2015 IEEE Conference on Computer Vision and Pattern Recognition (CVPR).

[40] Zhou B., Lapedriza A., Khosla A., Oliva A., Torralba A. (2018). Places: A 10 Million Image Database for Scene Recognition. IEEE Trans. Pattern Anal. Mach. Intell..

